# Supplementary material for: Excited-state nonadiabatic dynamics in explicit solvent using machine learned interatomic potentials
Source: Digit Discov. 2025 Apr 24;4(6):1478–91. doi: 10.1039/d5dd00044k (PMC12060776; doi:10.1039/d5dd00044k)
Supplement: DD-004-D5DD00044K-s001 [file DD-004-D5DD00044K-s001.pdf]

Supplementary Information (SI) for Digital Discovery.
This journal is © The Royal Society of Chemistry 2025

Supplementary Material: Excited-state nonadiabatic dynamics in explicit solvent using machine learned interatomic potentials

Maximilian X. Tiefenbacher,[a,b] Brigitta Bachmair,[a,b,c]
Cheng Giuseppe Chen,[d] Julia Westermayr,[e,f]
Philipp Marquetand,[a,c] Johannes C. B. Dietschreit,[‡c]
Leticia González[*a,c]

[a] Research Platform on Accelerating Photoreaction Discovery (ViRAPID),
University of Vienna,Währinger Straße 17, 1090 Vienna, Austria.
[b] Vienna Doctoral School in Chemistry,
University of Vienna, Währinger Straße 42, 1090 Vienna, Austria.
[c] Institute of Theoretical Chemistry, Faculty of Chemistry,
University of Vienna, Währinger Straße 17, 1090 Vienna, Austria.
[d] Department of Chemistry, Sapienza University of Rome, Piazzale Aldo Moro, 5, Rome, 00185, Italy.
[e] Wilhelm-Ostwald Institute, University of Leipzig, Linnéstraße 2, 04103 Leipzig.
[f] Center for Scalable Data Analytics and Artificial Intelligence (ScaDS.AI),
Dresden/Leipzig, Humboldtstraße 25, 04105 Leipzig, Germany.
‡ johannes.dietschreit@univie.ac.at
* leticia.gonzalez@univie.ac.at

Contents

1 Excitation analysis **S3**

2 Parity Plots **S4**

2.1 Performance on Set I S5
2.1.1 Split by Trajectory; 100 % of Available Data S5
2.1.2 Split by Trajectory; 33 % of Available Data S6
2.1.3 Random Split; 100 % of Available Data S7
2.1.4 Random Split; 33 % of Available Data S8
2.2 Performance on Set II S9
2.2.1 Split by Trajectory; 100 % of Available Data S9
2.2.2 Split by Trajectory; 33 % of Available Data S10
2.2.3 Split by Trajectory; 1 % of Available Data S11
2.2.4 Random Split; 100 % of Available Data S12

2.2.5 Random Split; 33 % of Available Data S13
2.2.6 Random Split; 1 % of Available Data S14
2.3 Performance on first 75 fs of Set II S15
2.3.1 Split by Trajectory; 100 % of Available Data S15
2.3.2 Split by Trajectory; 33 % of Available Data S16
2.3.3 Random Split; 100 % of Available Data S17
2.3.4 Random Split; 33 % of Available Data S18

3 Time Evolution of Averaged Properties S19
3.1 Electronic Populations S19
3.2 Average State Energy S22

4 Structural analysis of the dynamics S23
4.1 Ring opening S23
4.2 Hopping geometries S23
4.2.1 $S_1 \rightarrow S_0$ S23
4.2.2 $S_2 \rightarrow S_1$ S27

5 Additional Figures S29

References S30

1 Excitation analysis

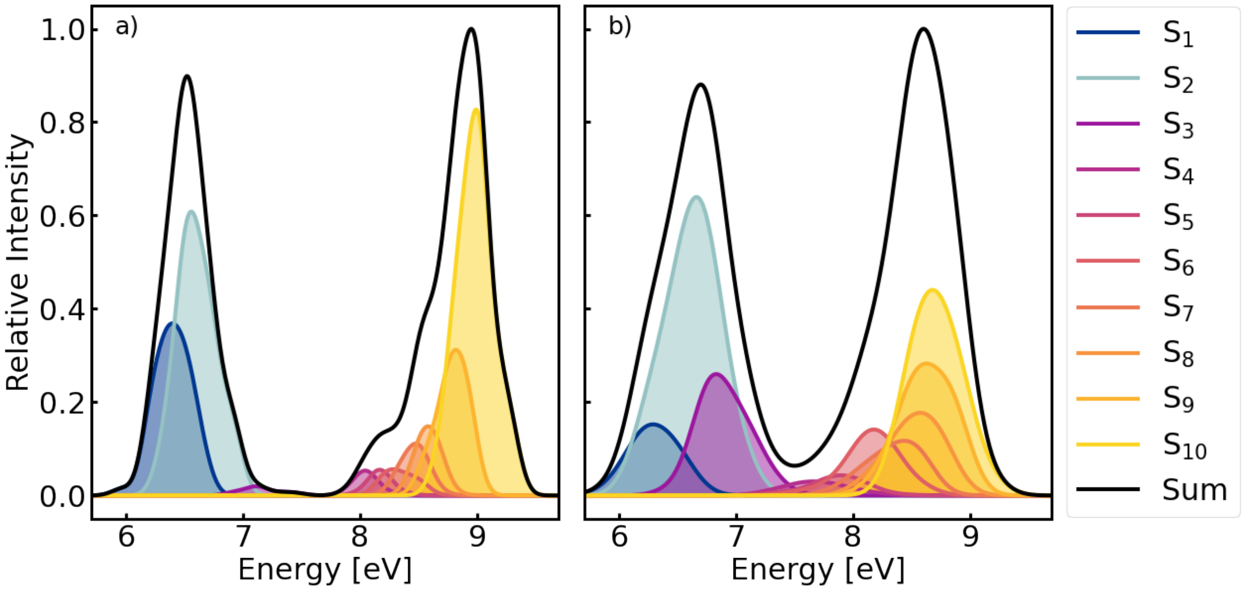

Figure S1: Absorption spectrum of furan based on the lowest 10 excited singlet states for a) solvated in explicit water obtained as average over 100 MM-MD snapshots and b) in the gas phase obtained from 100 Wigner samples.

Figure S1 shows the simulated spectra of furan solvated in water (a) and gas phase (b). While the two peaks of the total intensity are quite similar between the two spectra, the solvated furan shows slimmer peaks. Hence, in the case of the solvated furan the area between 7 and 8 eV is almost completely dark. This is mainly caused by the S_3 being a dark state in solution, while it contributes significantly to the signal in the gas phase. The S_1 shows the opposite behavior, having less intensity in the gas phase than in solution. However, the reduction is not as large. Furthermore, S_6 is also brighter in the gas phase, while S_{10} has a lower peak.

The peak between 6 and 7 eV is created by transition to S_1, S_2, and S_3, with S_2 having almost triple the height of the S_1 and S_3 in vacuum. In solution, the relative intensity of the transition to S_2 remains largely unchanged, however, S_1 increases in intensity and S_3 is an almost dark state.

Table S1 shows an analysis of the first ten excitations of furan in the gas phase. The first two excitations are $\pi \rightarrow \pi^*$ transitions. While the first excited state is dark for the equilibrium geometry, the second one is has the strongest oscillator strength. All other states with an oscillator strength of zero in this table are 0 because of selection rules, which are do not apply to distored geometries obtained in the Wigner sampling (Fig. S1b).

Table S1: TDA TD-DFT excitation energies, oscillator strengths, character of the transitions, and the dipole moment of the first 10 excited states are shown. Obtained for furan optimized in the gas phase using the BP86/def2-SVP.

State	Excitation energy [eV]	Oscillator strength [10^{-3}]	Character	Dipole moment [Debye]
S_0	-	-	-	0.19
S_1	6.49	0.0	$\pi \rightarrow \pi^*$	0.27
S_2	6.78	236.5	$\pi \rightarrow \pi^*$	0.19
S_3	7.24	0.0	$\pi \rightarrow$ ryd	0.93
S_4	8.13	0.0	$\pi \rightarrow$ ryd	0.42
S_5	8.16	4.2	$\pi \rightarrow$ ryd	1.46
S_6	8.37	1.0	n$\rightarrow \pi^*$	0.17
S_7	8.45	0.2	$\pi \rightarrow$ ryd	0.46
S_8	8.56	0.3	$\pi \rightarrow$ ryd	2.03
S_9	9.05	1.1	$\sigma \rightarrow \pi^*$	0.11
S_{10}	9.22	0.0	$\sigma \rightarrow \pi^*$,$\pi \rightarrow$ ryd	0.75

2 Parity Plots

In this section, we have collected the parity plots for all trained models. The plots show the scatter of predicted vs. ground-truth label for energies and forces of the five electronic states predicted by all models as well as the indirectly predicted energy gap between neighboring levels (four differences). The gap is not a direct output of the models, but rather the difference of two adjacent energy levels.

The first subsection shows the model performance on the original test set, i.e. frames taken from Set I (training and validation data is always taken from this set). We only show parity plots for models trained on 100 % and 33 % of the data, as when retaining only 1 % of the data, the parity plots are almost empty.

In contrast, the tests performed on Set II always use exactly the same frames. Therefore, the number of points does not change with the type of split or the amount of subsampling used during the training.

2.1 Performance on Set I

2.1.1 Split by Trajectory; 100 % of Available Data

Model 1

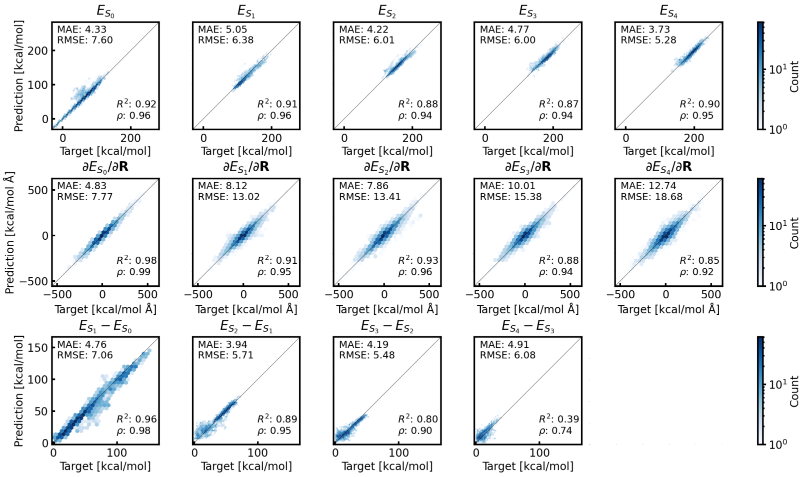

Model 2

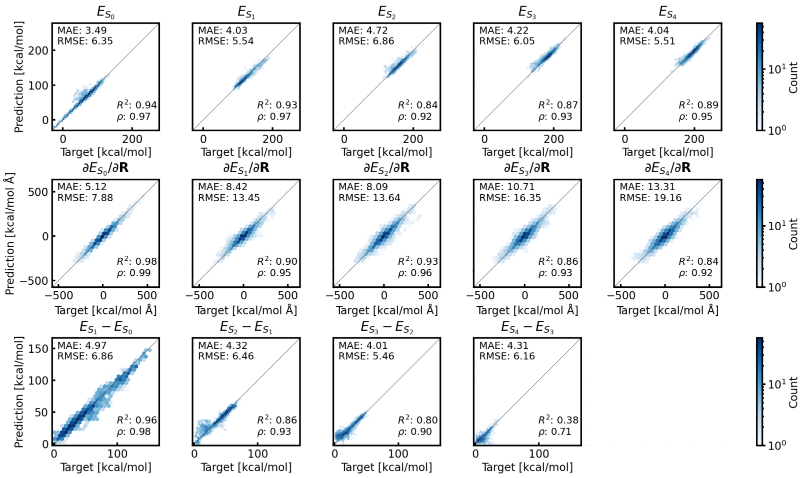

Model 3

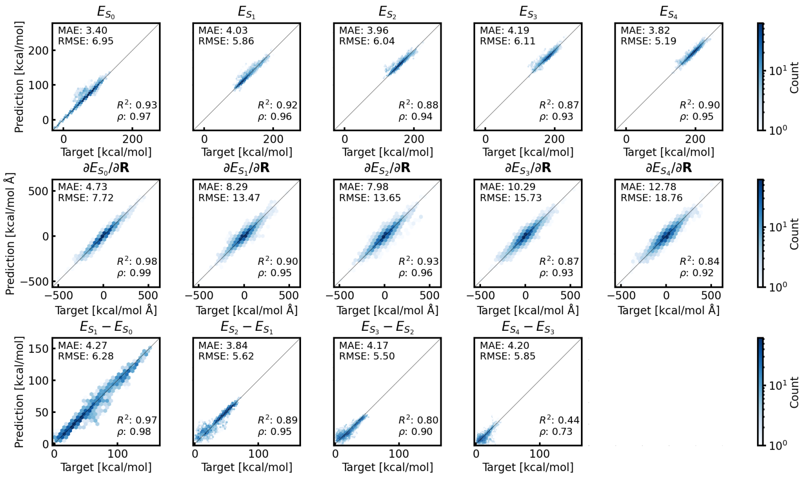

Figure S2: Parity plots for the set I test set for the three "Split by Trajectory" models trained on 100 % of the available frames (every 0.5 fs) from the 36 training trajectories.

2.1.2 Split by Trajectory; 33 % of Available Data

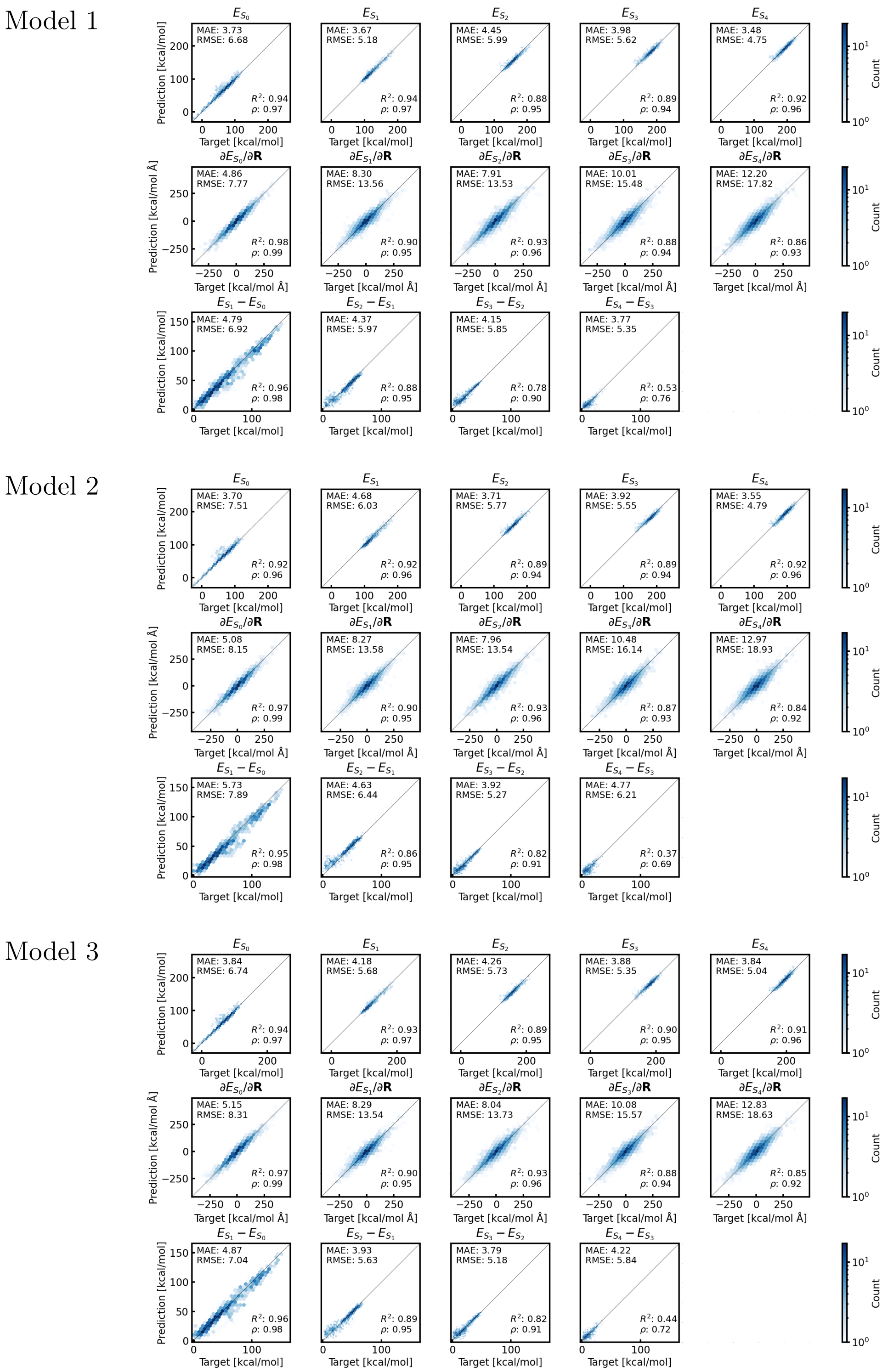

Figure S3: Parity plots for the set I test set for the three "Split by Trajectory" models trained on 33 % of the available frames (every 1.5 fs) from the 36 training trajectories.

2.1.3 Random Split; 100 % of Available Data

Model 1

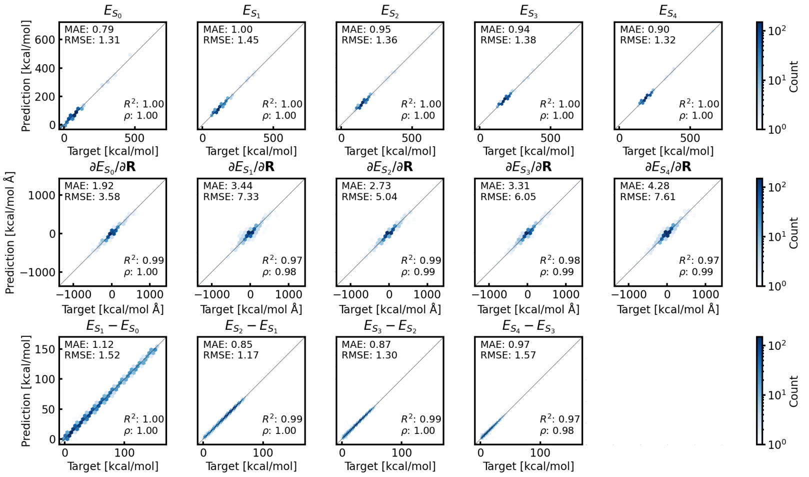

Model 2

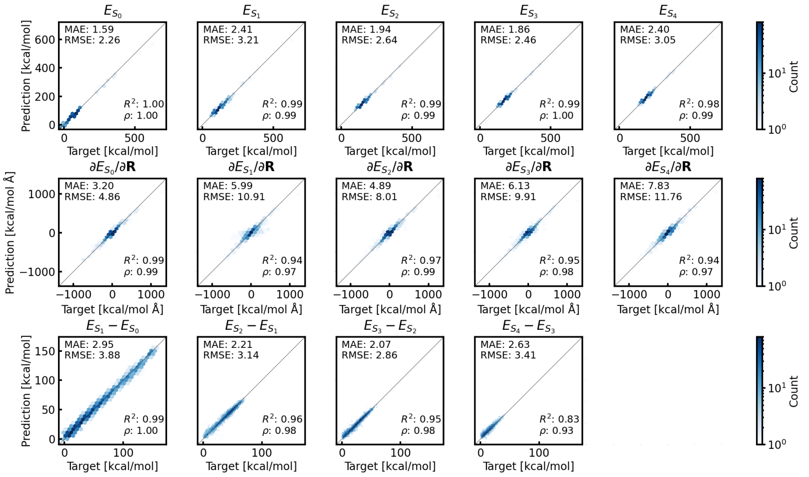

Model 3

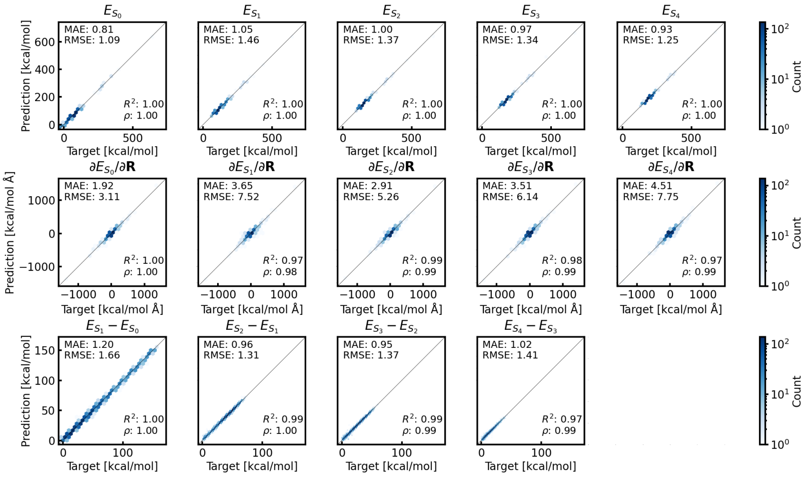

Figure S4: Parity plots for the set I test set for the three "Random Split" models trained on 100 % of the available frames (every 0.5 fs) from the 36 training trajectories.

2.1.4 Random Split; 33 % of Available Data

Model 1

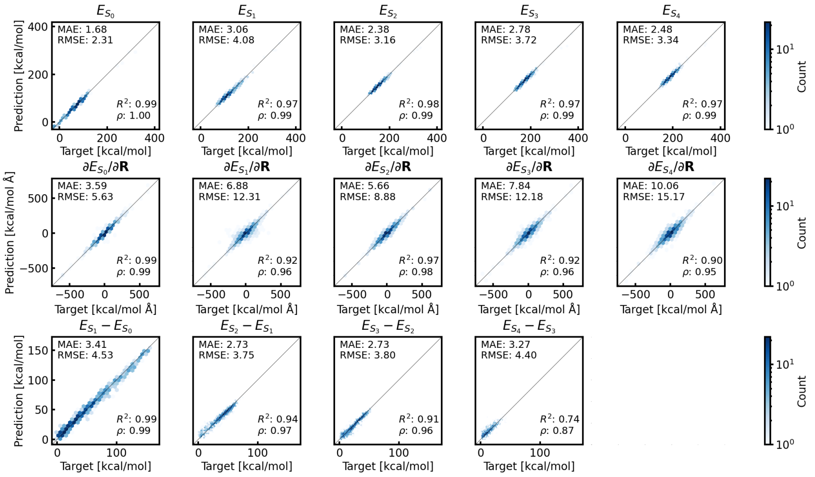

Model 2

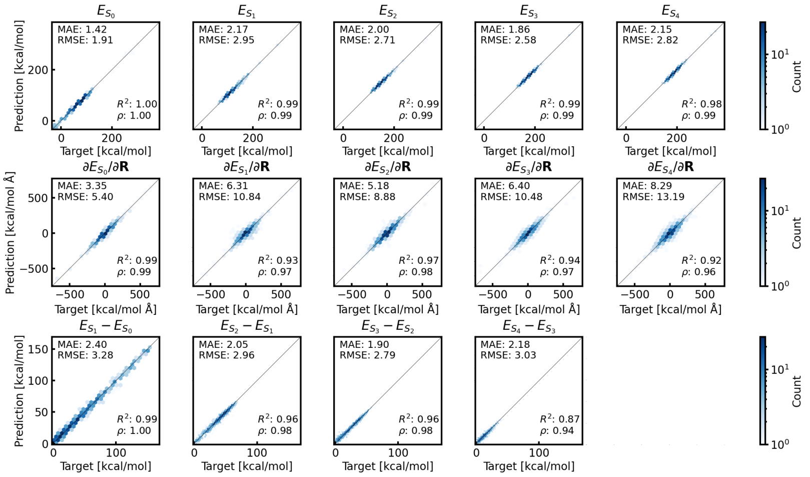

Model 3

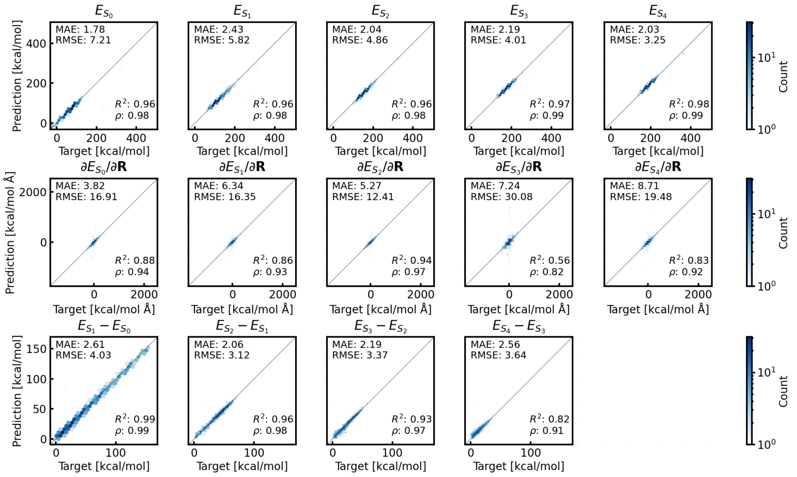

Figure S5: Parity plots for the set I test set for the three "Random Split" models trained on 33 % of the available frames (every 1.5 fs) from the 36 training trajectories.

2.2 Performance on Set II

2.2.1 Split by Trajectory; 100 % of Available Data

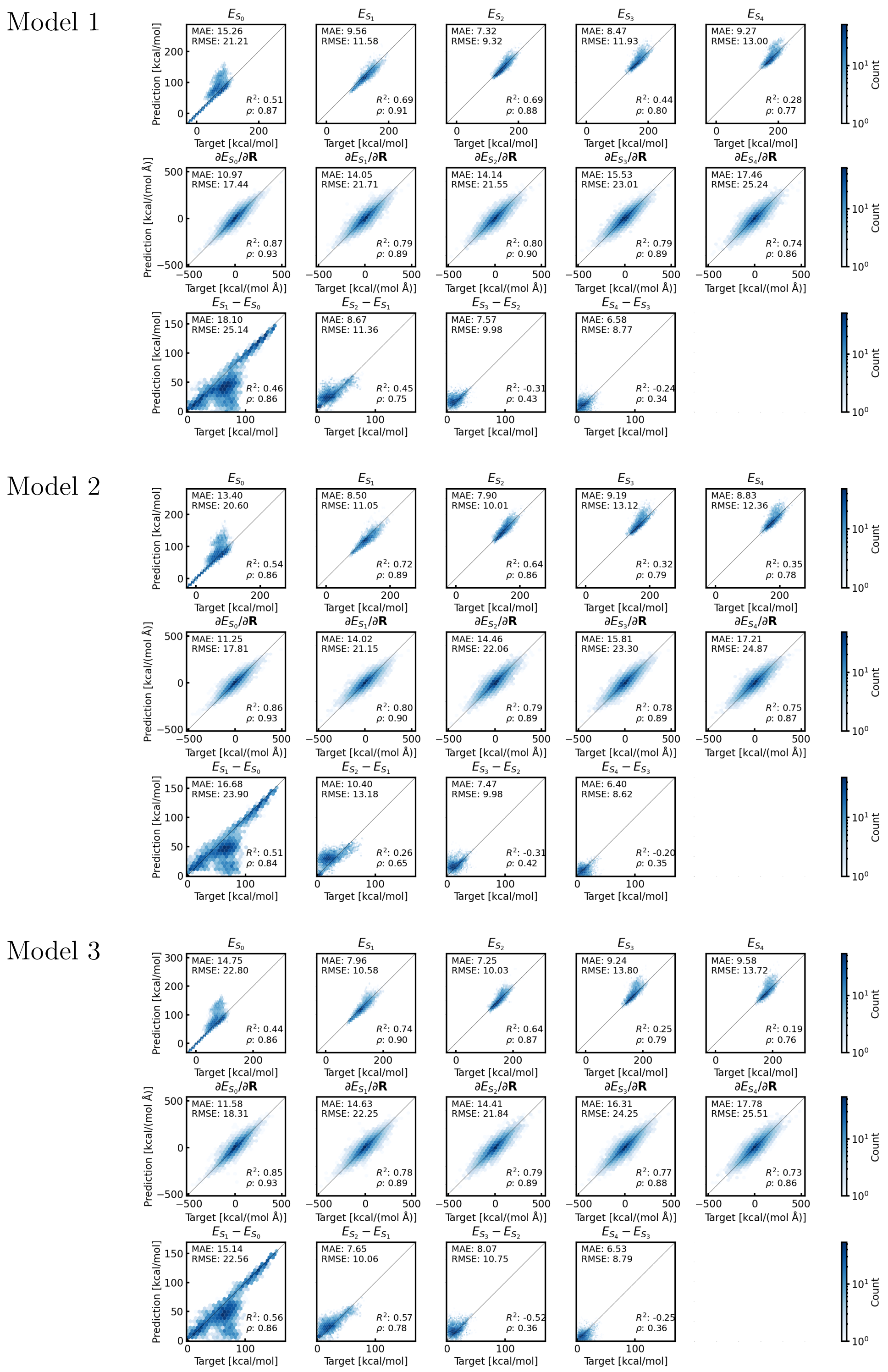

Figure S6: Parity plots on frames from Set II for the three "Split by Trajectory" models trained on 100 % of the available frames (every 0.5 fs) from the 36 training trajectories.

2.2.2 Split by Trajectory; 33 % of Available Data

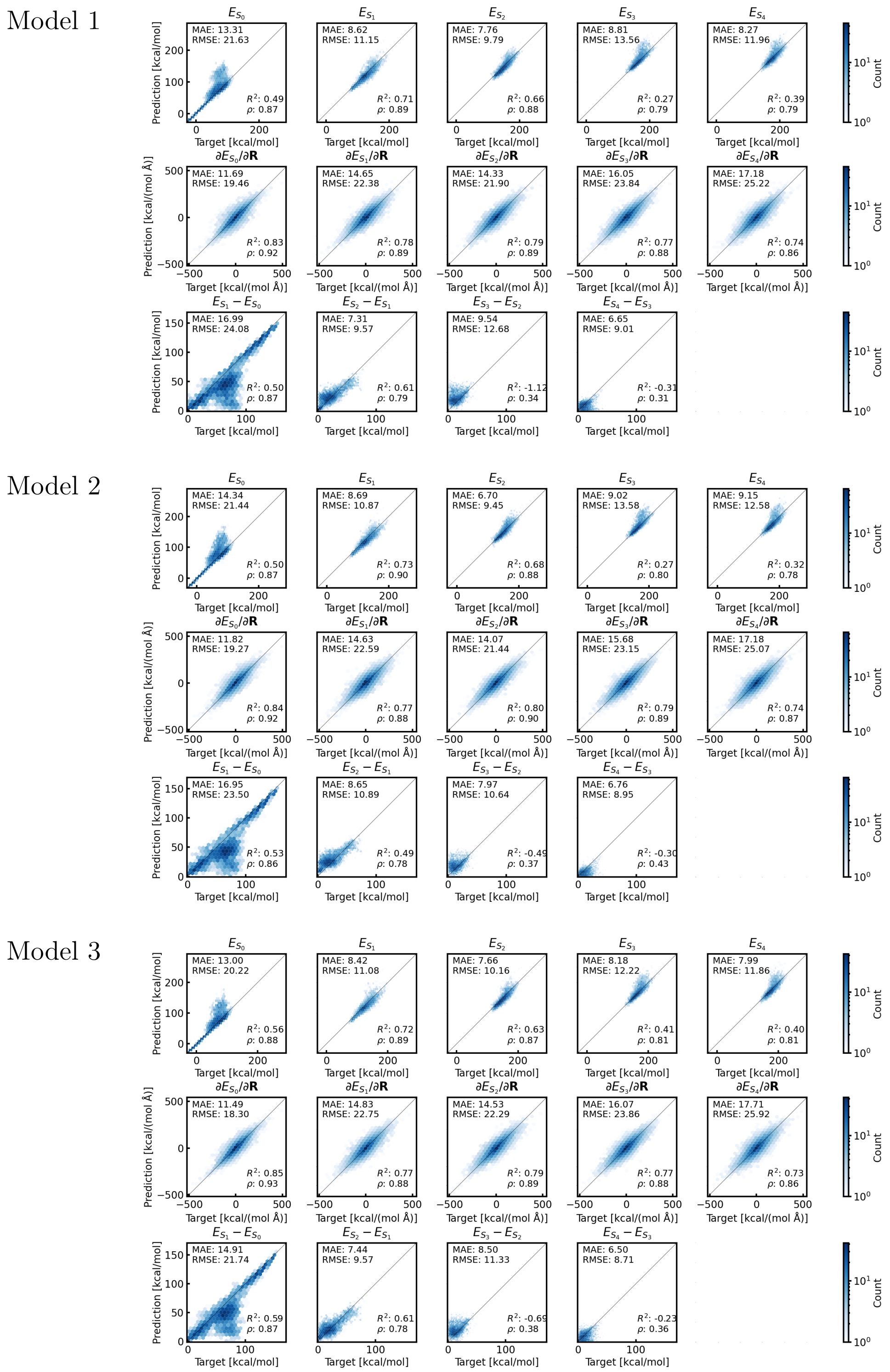

Figure S7: Parity plots on frames from Set II for the three "Split by Trajectory" models trained on 33 % of the available frames (every 1.5 fs) from the 36 training trajectories.

2.2.3 Split by Trajectory; 1 % of Available Data

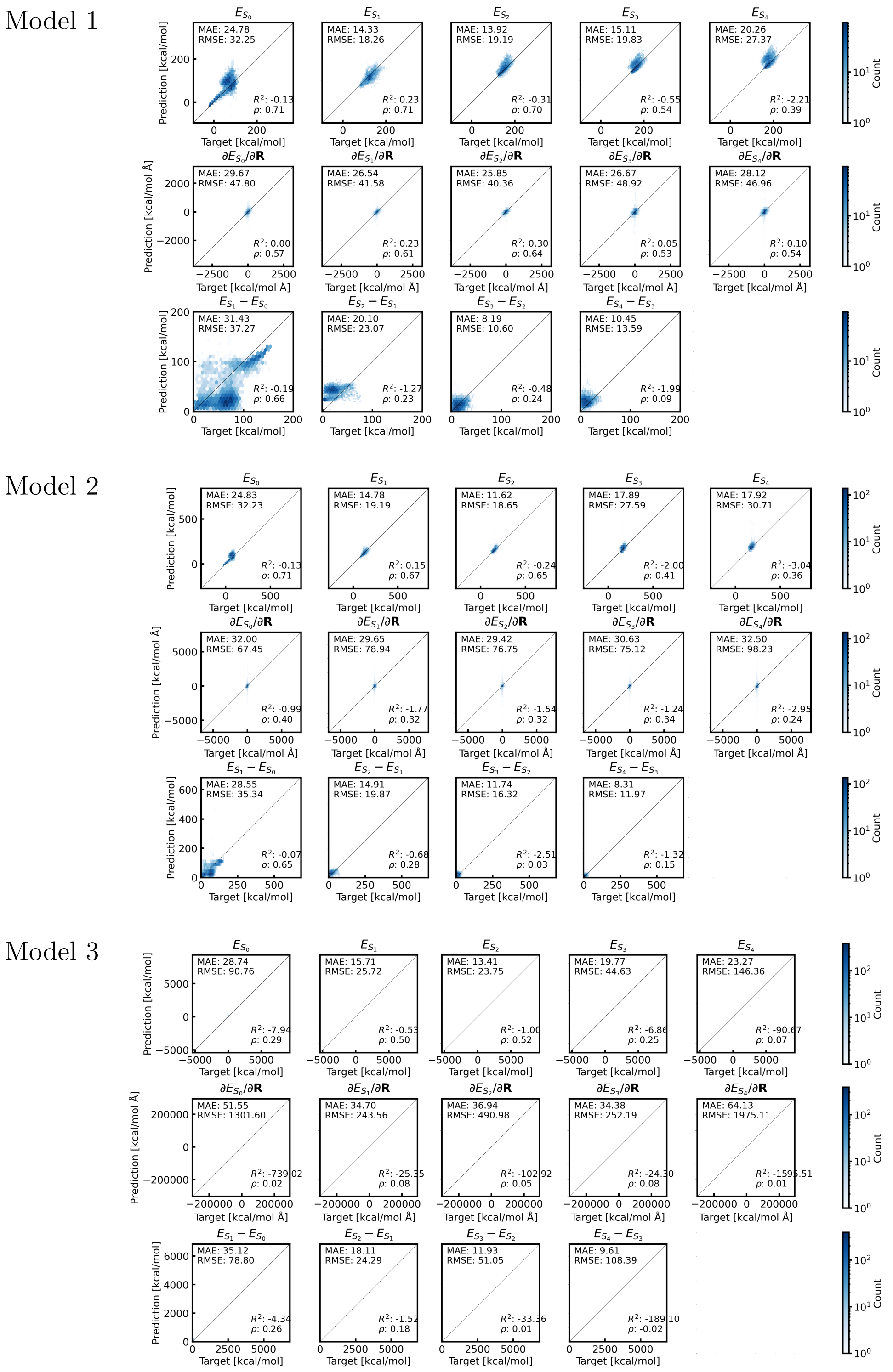

Figure S8: Parity plots on frames from Set II for the three "Split by Trajectory" models trained on 1 % of the available frames (every 50 fs) from the 36 training trajectories.

2.2.4 Random Split; 100 % of Available Data

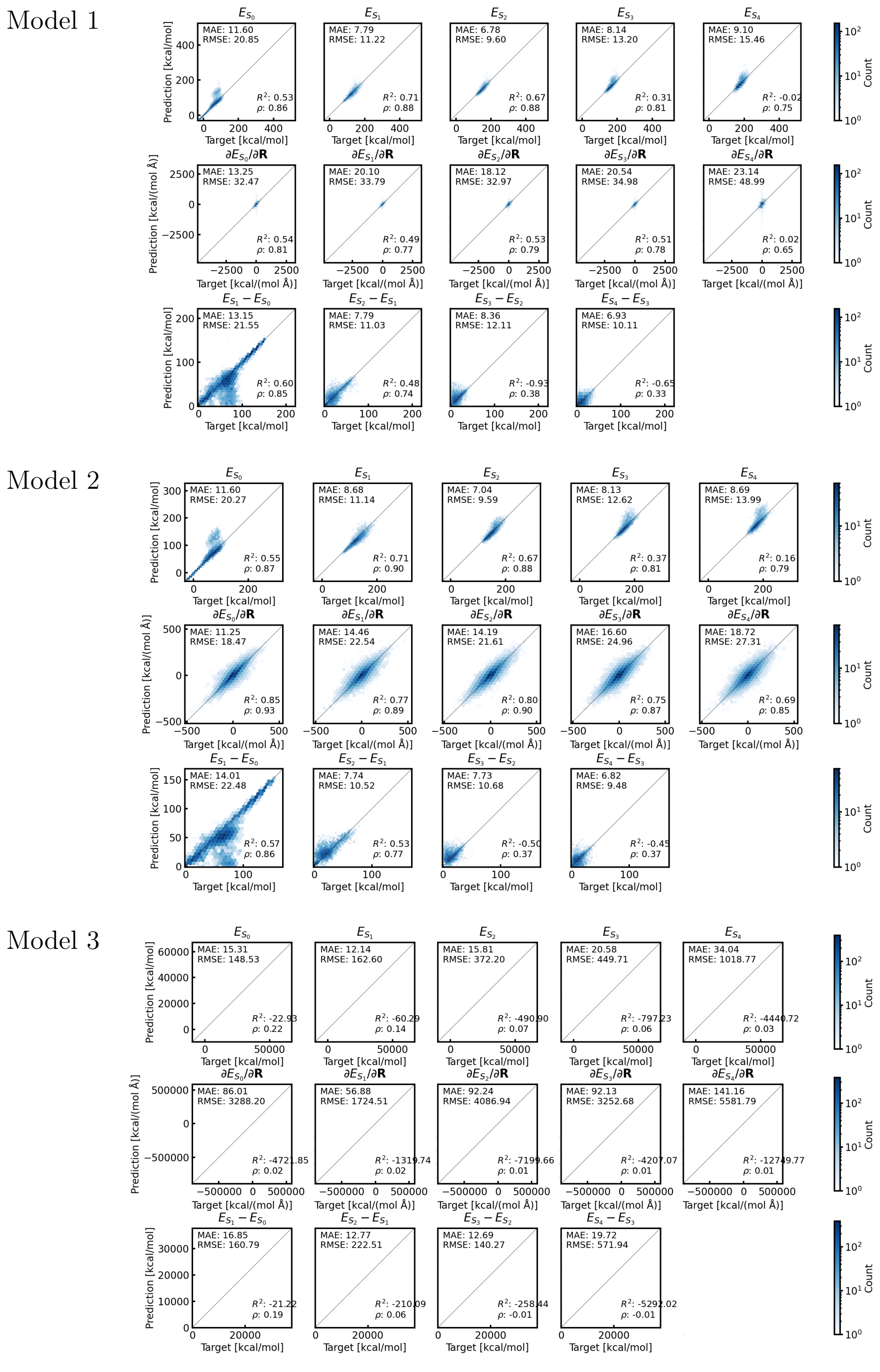

Figure S9: Parity plots on frames from Set II for original test for the three "Random Split" models trained on 100 % of the available frames (every 0.5 fs) from the 36 training trajectories.

2.2.5 Random Split; 33 % of Available Data

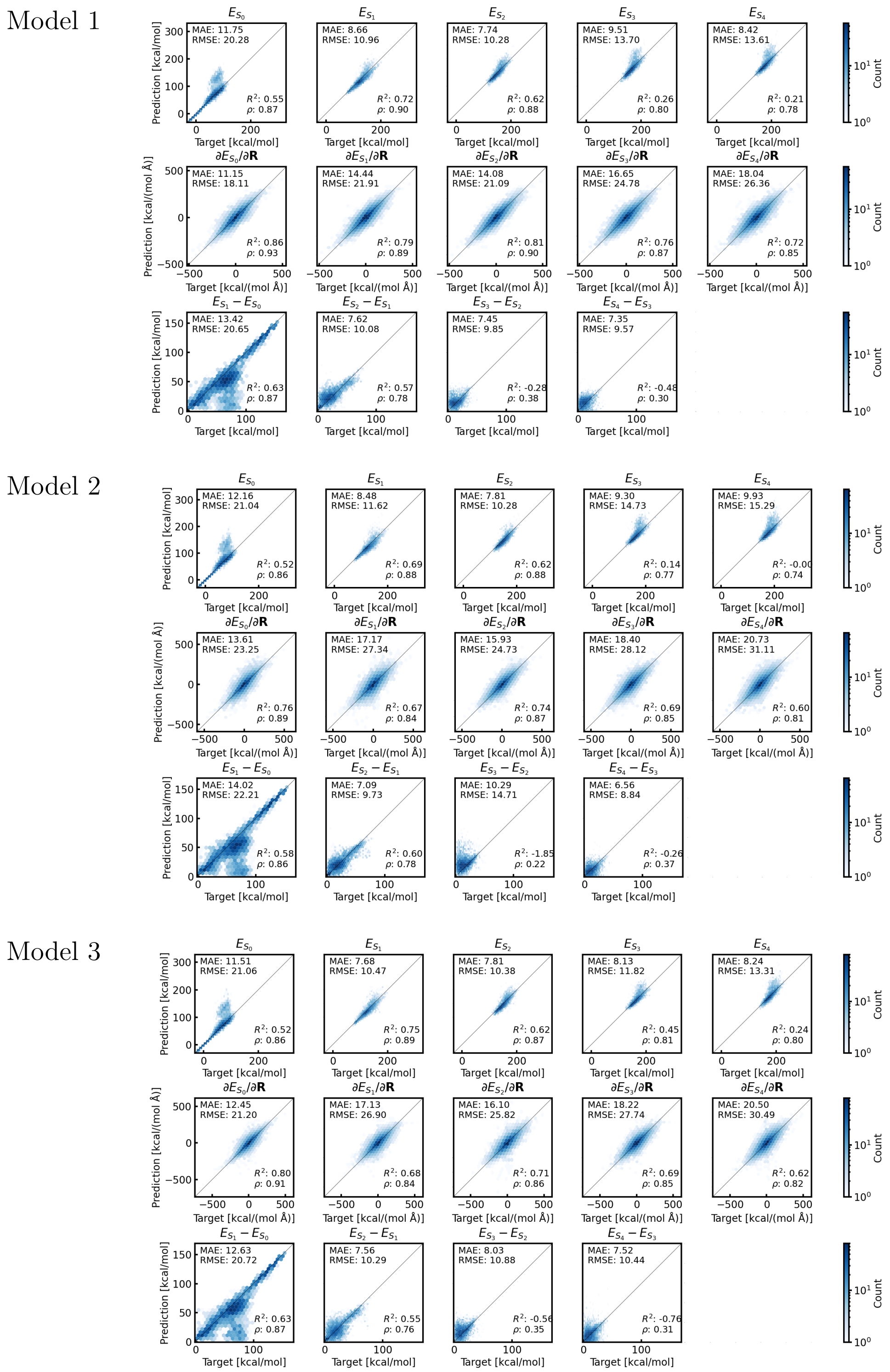

Figure S10: Parity plots on frames from Set II for original test for the three "Random Split" models trained on 33 % of the available frames (every 1.5 fs) from the 36 training trajectories.

2.2.6 Random Split; 1 % of Available Data

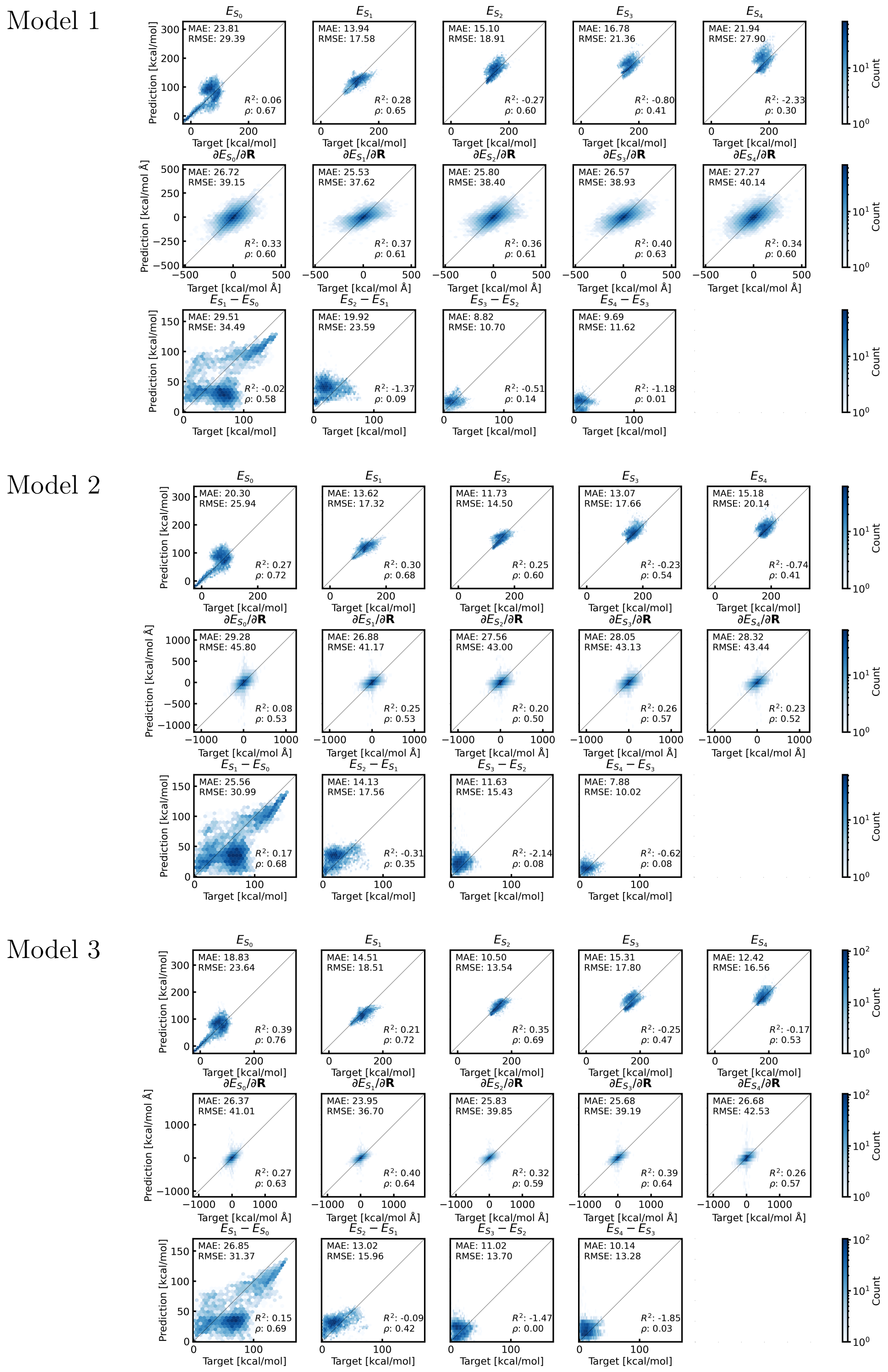

Figure S11: Parity plots on frames from Set II for original test for the three "Random Split" models trained on 1 % of the available frames (every 50 fs) from the 36 training trajectories.

2.3 Performance on first 75 fs of Set II

2.3.1 Split by Trajectory; 100 % of Available Data

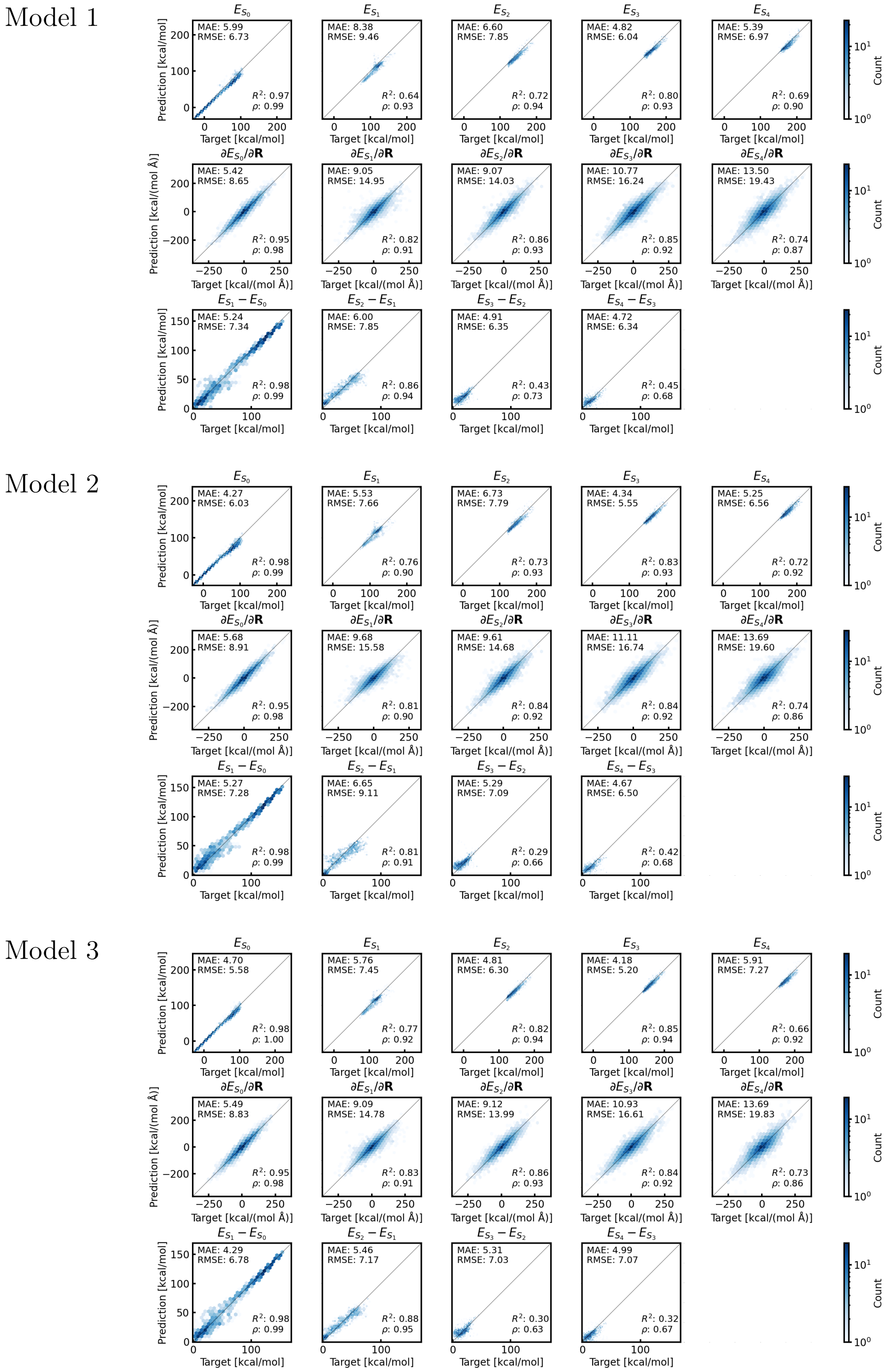

Figure S12: Parity plots on frames from the first 75 fs of trajectories from Set II for the three "Split by Trajectory" models trained on 100 % of the available frames (every 0.5 fs) from the 36 training trajectories.

2.3.2 Split by Trajectory; 33 % of Available Data

Model 1

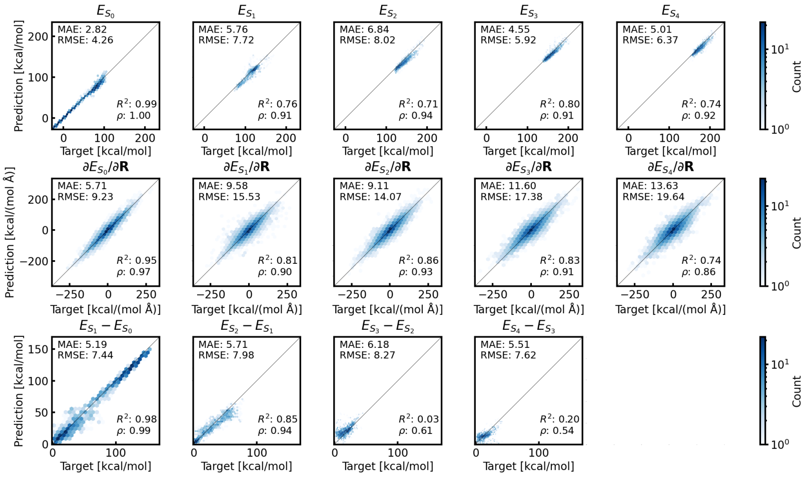

Model 2

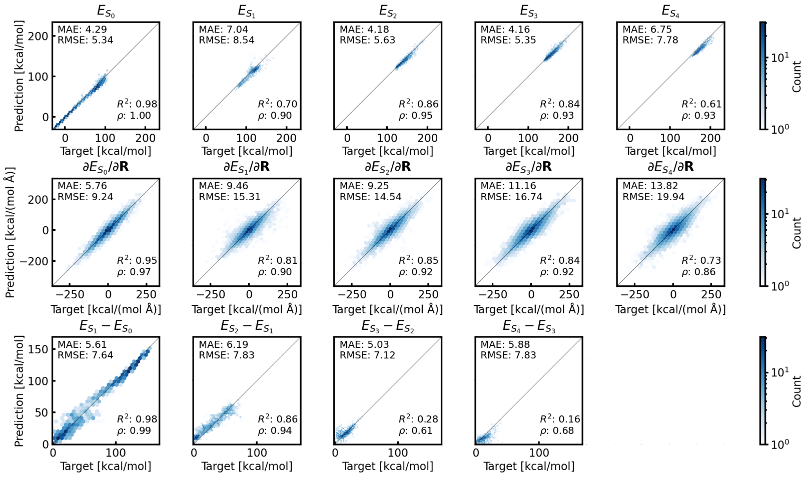

Model 3

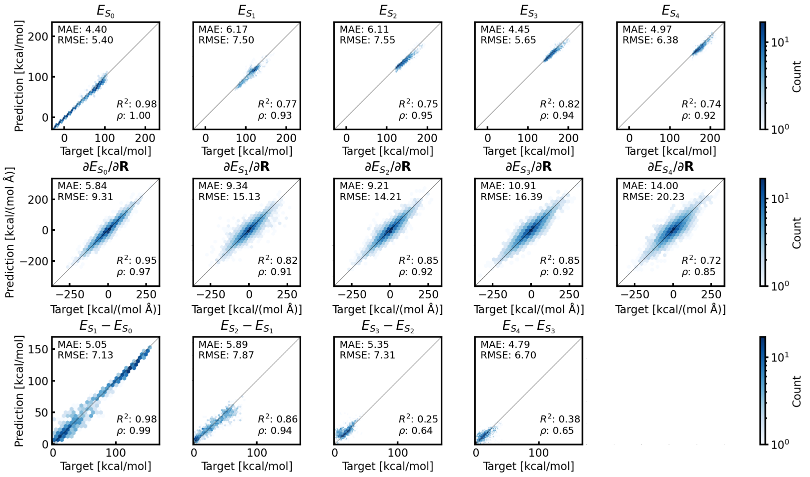

Figure S13: Parity plots on frames from the first 75 fs of trajectories from Set II for the three "Split by Trajectory" models trained on 33 % of the available frames (every 1.5 fs) from the 36 training trajectories.

2.3.3 Random Split; 100 % of Available Data

Model 1

Model 2

Model 3

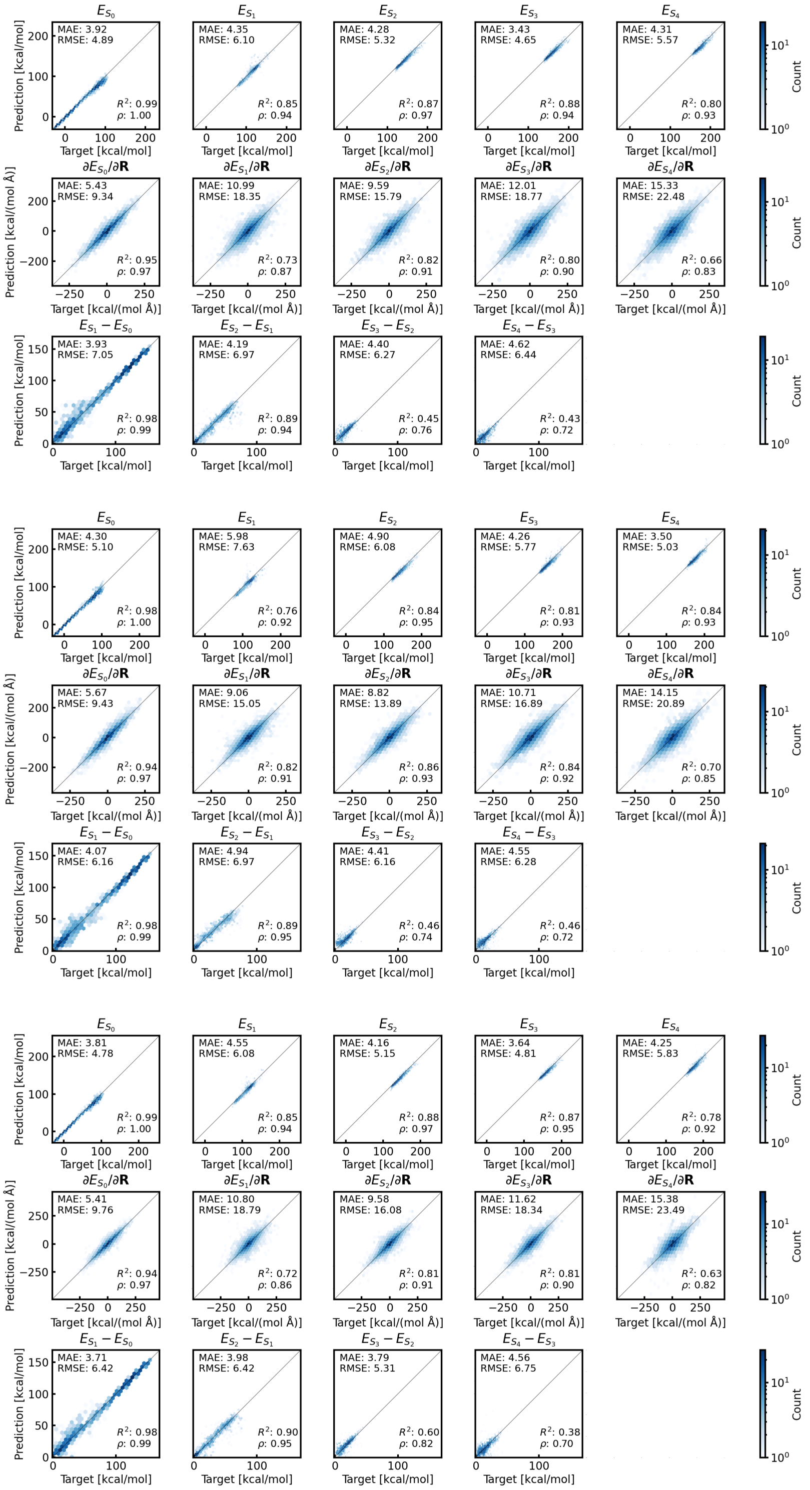

Figure S14: Parity plots on frames from the first 75 fs of trajectories from Set II for original test for the three "Random Split" models trained on 100 % of the available frames (every 0.5 fs) from the 36 training trajectories.

2.3.4 Random Split; 33 % of Available Data

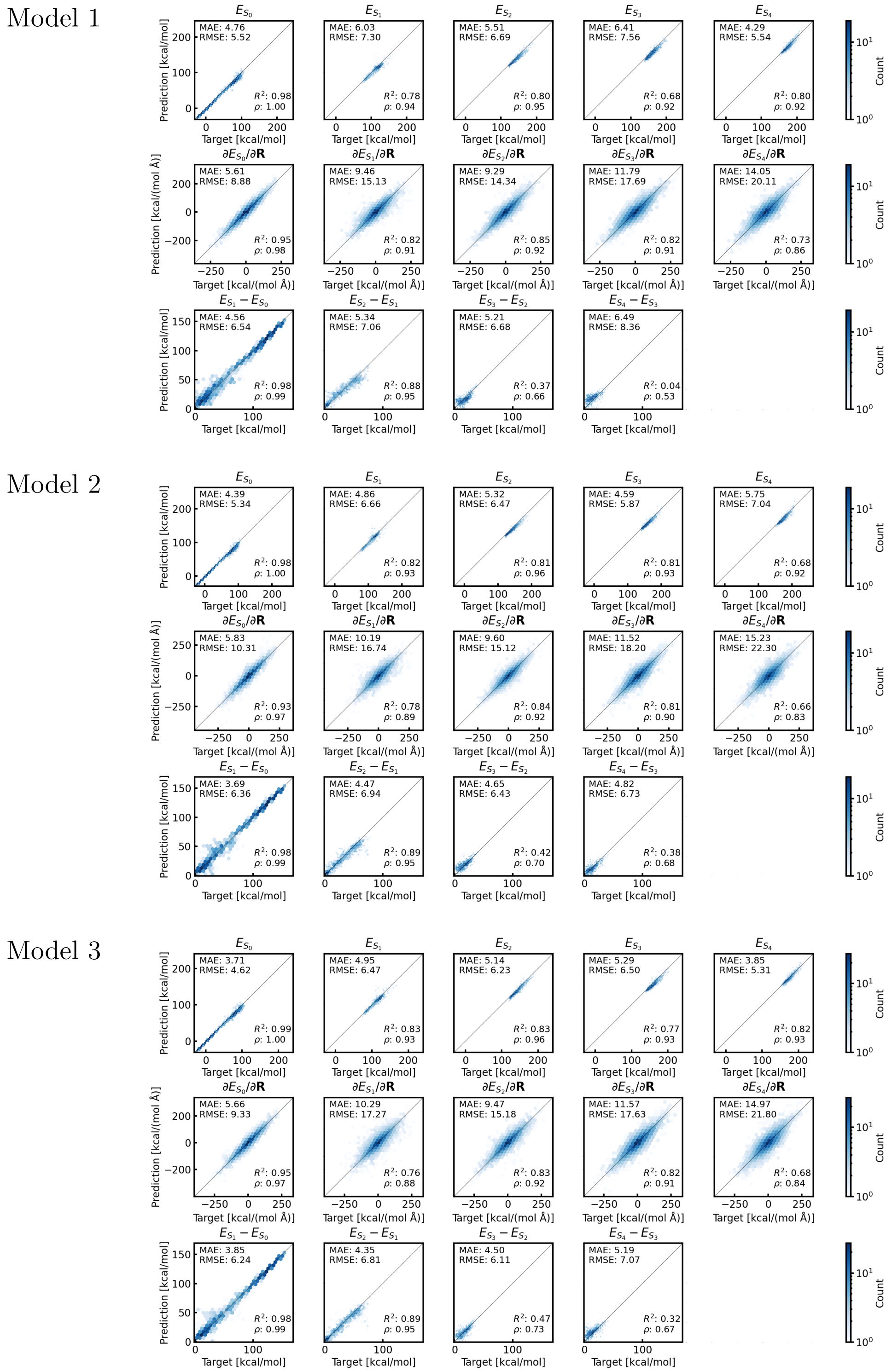

Figure S15: Parity plots on frames from the first 75 fs of trajectories from Set II for original test for the three "Random Split" models trained on 33 % of the available frames (every 1.5 fs) from the 36 training trajectories.

3 Time Evolution of Averaged Properties

3.1 Electronic Populations

In this section, we show the average electronic population derived from the active state for all ML/MM simulations (including those not shown in the main text). These populations are the basis used to compute the relaxation times reported in the manuscript. All of these trajectories were performed with the initial conditions from set II. The electronic populations of the reference QM/MM dynamics (set II) are shown as dashed lines. As a reminder, we trained 18 different models altogether: We compared two different ways to split the training data set (set I), namely "Random Split" and "Split by Trajectory" and additionally we used three different training set sizes obtained by subsampling the trajectories (100 %, 33 % and 1 % of the data). For each combination of split and subsampling we trained three independent models, i.e. with different random weight initializations.

Figure S16 shows the results for the "Split by Trajectory" models. The 100 % models do not agree well with the QM/MM simulations, which can also be seen in the corresponding half-lives (see Table 1 in the main text). The S_2 decays too slowly (except for model no. 2) and the transitions to the ground state are also much slower than in the reference. The 33 % models perform better than the 100 %, those were also shown in the main text.

For the 33 % models, the agreement increases from model no. 1 to 3. Model no. 1, produces dynamics with a very slow decay from the S_2 into S_1 so that the population of the S_1 appears constant as the two decay rates are similar (incorrectly so). Model two produces dynamics that are qualitatively correct, only the relaxation times are too large (compare Table 1 in the main text). Model three performs the best, the intersections of the population curves occur at almost the same time after excitation as in the QM/MM simulations. The models trained with only 1 % of the data show very poor performance, which was expected based on the low amount of available data.

Figure S17 shows the results from "Random Split" procedure. As has been mentioned in the manuscript, the occupations for models one and three with 100 % of the data agree quite well with the QM/MM simulations. They both only slightly overestimate the relaxation times and also show very similar qualitative agreement. Model two on the other hand shows quite bad agreement strongly overestimating the relaxation times leading to qualitative and quantitative disagreement. The models for 33 % of the data perform quite similar from a qualitative point of view. All of them have electronic population staying too long the S_2, lading to slower over all relaxation times to the S_0. Model 2 seems to perform the best, which is also supported by the computed half-life times. As expected, the models with only 1 % of the data show very large deviations as was the case for the "Split by Trajectory" models.

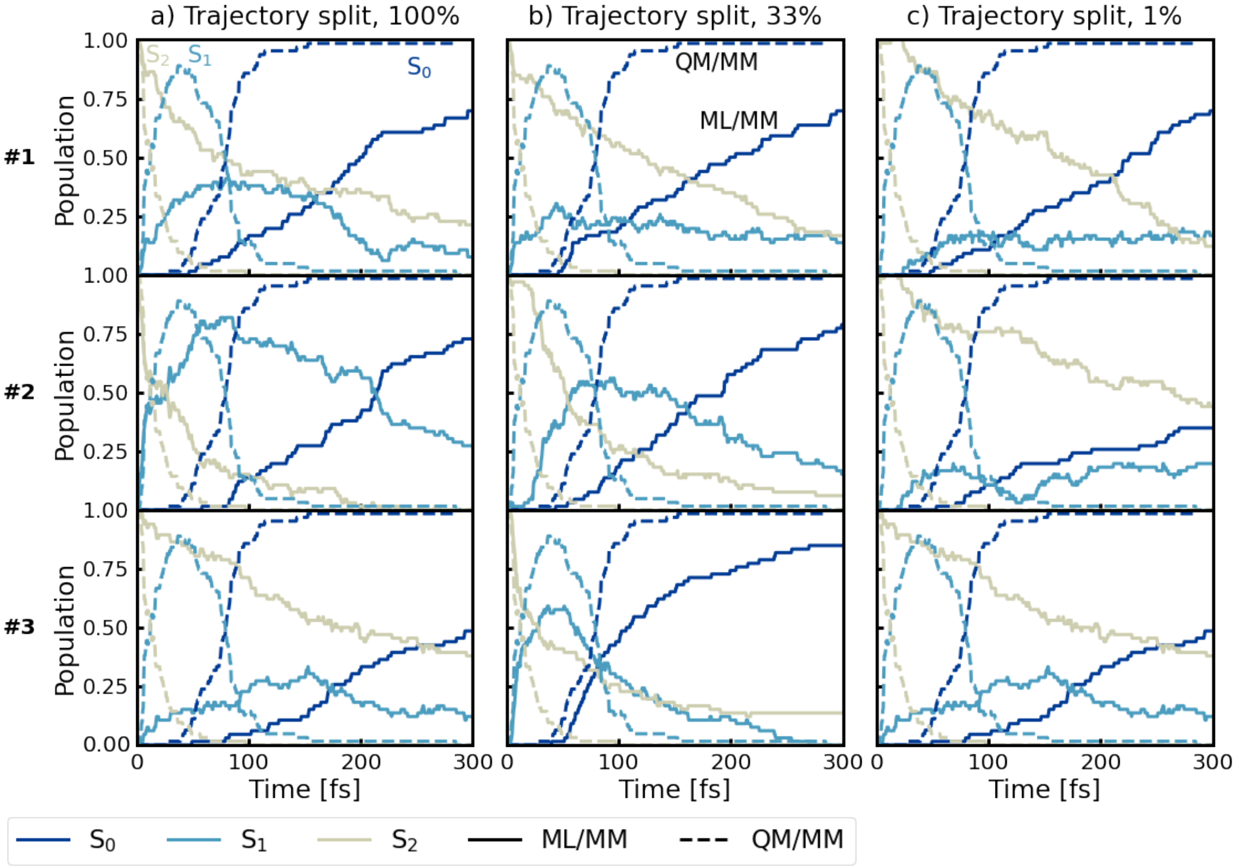

Figure S16: Electronic populations based on the active state with the initial conditions from Set II. The potentials for these populations were obtained from models trained with the "Split by Trajectory" procedure with 100 %, 33 % and 1 % of the data. For each of the split three different weight initializations were used resulting in a total of nine models.

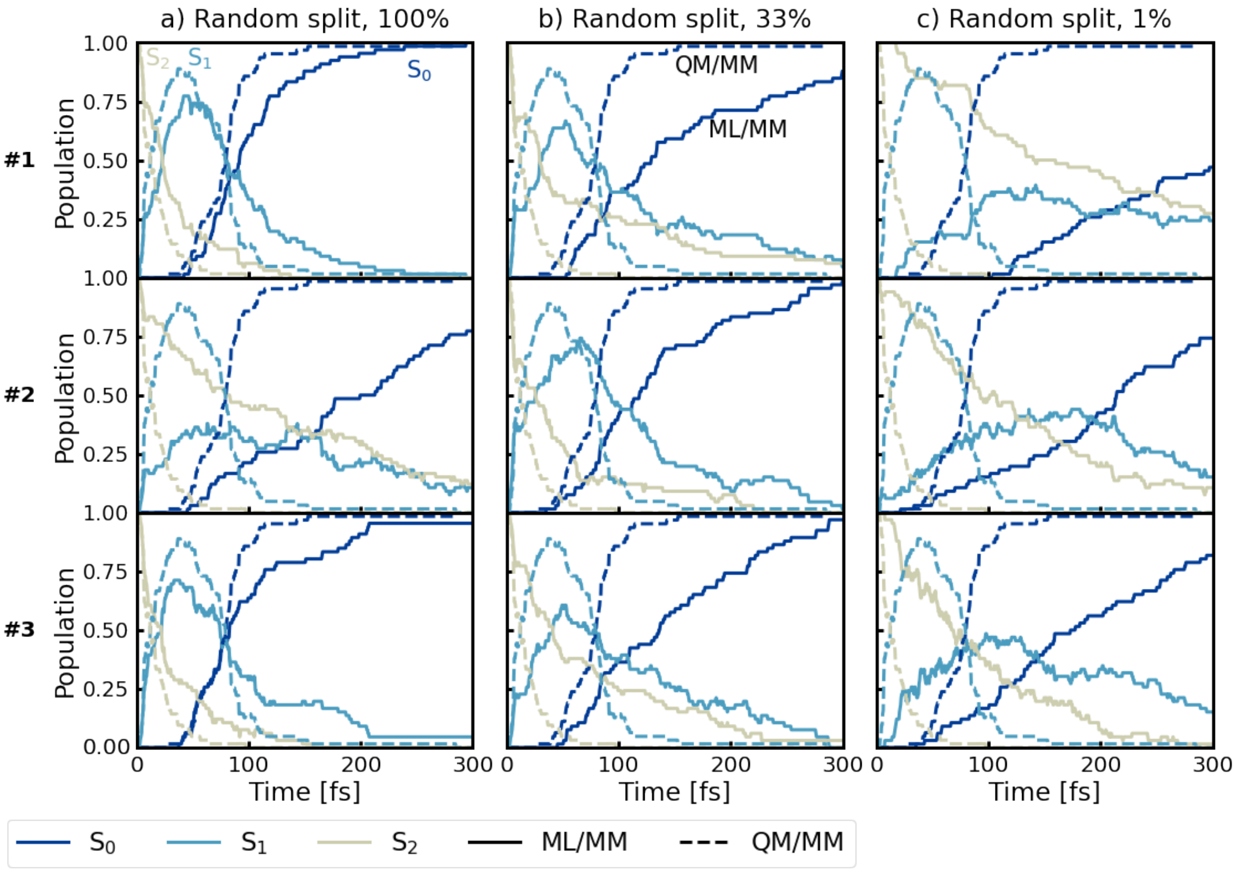

Figure S17: Electronic populations based on the active state with the initial conditions from Set II. The potentials for these populations were obtained from models trained with the "Random split" procedure with 100 %, 33 % and 1 % of the data. For each of the split three different weight initializations were used resulting in a total of nine models.

3.2 Average State Energy

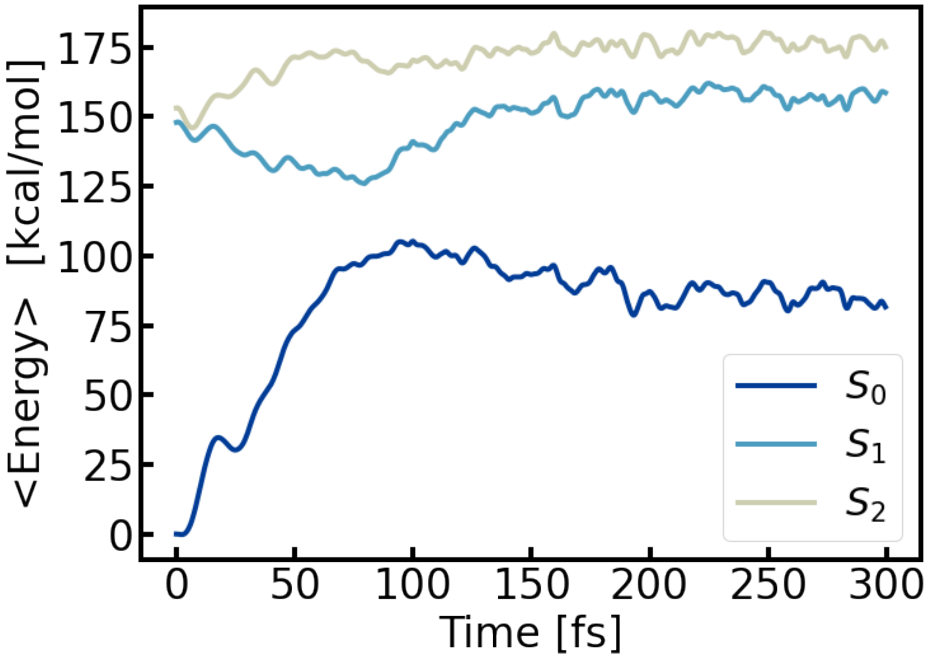

Figure S18: Time evolution of the average state energy. The averages were performed over all trajectories in set II. The average ground state energy at time step $t = 0$ was set to zero.

Figure S18 shows the evolution in time of the state energies averaged over all trajectories from QM/MM set II. The three values at $t = 0$ represent the average energy of each state for the configuration at the beginning of each surface hopping trajectory. The ground state has the lowest value at the beginning of the simulation, since the initial conditions were drawn from a ground-state MD. S_2 and S_1 are also very close at the beginning and basically touch after a few femtoseconds. This is reasonable as most trajectories relax very quickly from the second to the first excited state ($\tau_{2\to1} = 17$ fs, Table 1 in the main text). The energy of the ground state rapidly increases by 100 kcal/mol over the next 75 fs while furan descends in energy on the S_1 potential energy surface. The average state energies of S_1 and S_0 are the closest at the 75 fs mark, at which point roughly 50 % of the electronic population has been transferred back to the S_0 (see dashed lines in any subplot of Figures SS16 and SS17). The average S_0 energy then slowly decreases (while the other two states increase), likely transferring kinetic energy to the environment.

It is those configurations occurring after 75 fs that all models fit poorly as indicated by comparison of the parity plots showing all of set II (Sections S2.2) and only those configurations before 75 fs (Section S2.3). The transition to the electronic ground state induces the breakage of a carbon-oxygen bond, which is likely poorly described by DFT and in turn not well fitted by the FieldSchNet models.

4 Structural analysis of the dynamics

In this section, we analyze the structural changes during the dynamics simulations.

4.1 Ring opening

We start with an analysis of the distance between the oxygen atom and the neighboring carbon atoms (C_1 and C_4, see Fig. 1 in the main text) in the QM/MM simulations. By taking the maximum distance of these two bonds we monitor whether the furan ring opens up over the course of the simulation. We will refer to this distance as "maximal C-O distance" from now on.

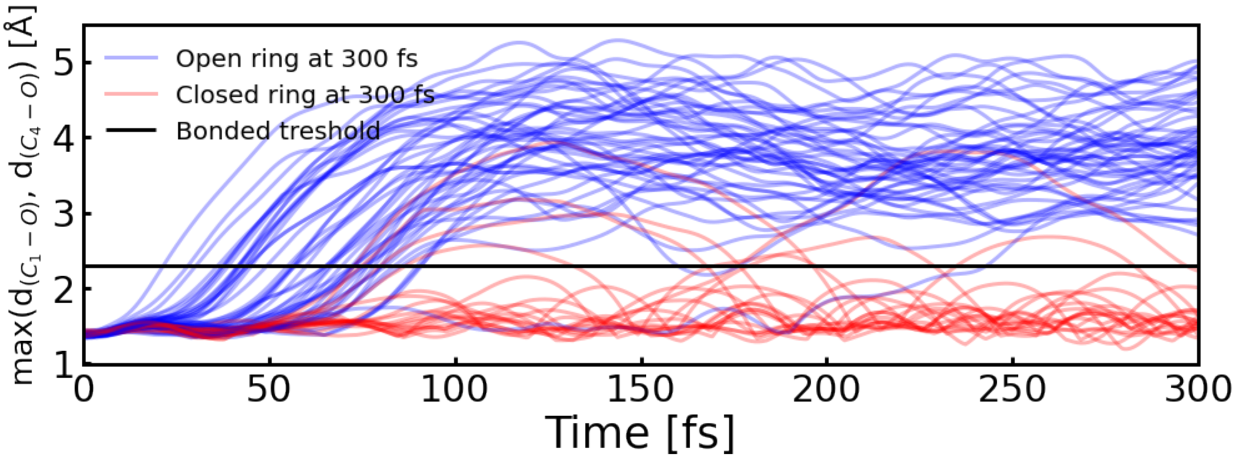

Figure S19: Maximum of C_1-O and C_4-O distances (see Fig. 1 of the main text for naming convention) over time for all QM/MM trajectories from set II. The red colored curves have a closed ring after the 300 fs simulation time. Trajectories are colored blue if the furan ring is open at the end of the simulation. We consider a ring closed if the maximum distance is smaller than 2.3 Å.

Figure S19 shows the maximum of the C_1-O and C_4-O distances over time for each trajectory in set II. We define a ring as open, when the maximum C-O distance is greater than 2.3 Å. Most of the ring openings occur soon after the excitation within the first 70 fs. In total, 53 out of 65 trajectories (ca. 82 %) display a ring opening (maximum of the two C-O distances above 2.3 Å). Six of them revert back to a closed configuration within the 300 s simulation time. One furan ring opens up at ca. 250 fs, while all other 49 trajectories do so much earlier. The remaining 12 trajectories do not exhibit any ring opening.

Fuji et al.[1] reported two relaxation channels, one in which the furan remains intact and the other in which one of the C-O bonds breaks. Our simulations recover both of these pathways.

4.2 Hopping geometries

Here we investigate the hopping geometries (the configurations of furan at the time step when the system switches PESs), and compare those found in the QM/MM and ML/MM simulations.

4.2.1 $S_1 \rightarrow S_0$

First, we focus on transitions between S_1 and S_0. In total, we obtain 88 hopping geometries between these two states (including hops in both directions). Based on our hopping scheme, hops from the ground state to higher states are not allowed, however, in regions where ground and excited state are

close or fully degenerate the first excited state can have a lower energy than the ground state in TDA TD-DFT leading to additional hops, which is not a problem, as this only occurs for configurations very close to the intersection seam.

When analyzing the hopping geometries, we solely inspect the configuration of furan. We describe the structures only with intramolecular distances by using the coulomb matrix[2] as defined in equation S1, this removes the influence of rotations and translations.

$$C_{ij} = \begin{cases} i = j & 0.5Z_i^{2.4} \\ i \neq j & \frac{Z_i Z_j}{|\mathbf{R}_i - \mathbf{R}_j|} \end{cases} \tag{S1}$$

where Z_x and $\mathbf{R}_x$ are the nuclear charge and position of atom x. To facilitate comparison between QM/MM and ML/MM hopping geometries, we perform a dimensionality reduction with principal component analysis (PCA). Since the Coulomb matrix is symmetric, we only used the values of the upper triangle as input for the PCA.

The first and second principle components (PCs) represent 88.8 % and 9.2 % of the variance of data, with all other PCs comprising less than 2 % together. Figure S20a shows the projection of the QM/MM hopping geometries onto the first (PC1) and second (PC2) principal component. The hopping geometries appear to be symmetric with respect to PC1. Further analysis of PC1 shows that it is mainly a linear combination of the two C-O bonds (C_1-O and C_4-O). PC2 is constructed by a linear combination of several non-hydrogen interatomic distances. When replacing PC1 with the maximum of these two bond distances, we obtain a clear diagonal line, see Fig. S20c.

When projecting the 66 hopping geometries from the ML/MM simulation with model no. 1 from the random split procedure with 100 % of the data onto the same principal components, we obtain Figure S20b. Analogous to Figure S20c, PC1 is substituted by the maximal C-O distance in Figure S20d.

Figure SS21 shows the projection of all hopping geometries between states S_1 and S_0 from all ML/MM simulations onto the maximal C-O distance and the PC2 from QM/MM PCA. The line fitted to the QM/MM reference data is included in every subplot to guide the eye. In the QM/MM ground truth the samples are scatter relatively equally around this line. In Figure SS21 we observe that some models tend to hop at significantly larger C-O distances or produce more clustered sets of hopping geometries, indicating that the fitted PES did not reproduce the same seam. In general, the more similar the projection of the hopping geometries for a model is the better the dynamics seems to reproduce the QM/MM ground truth as well.

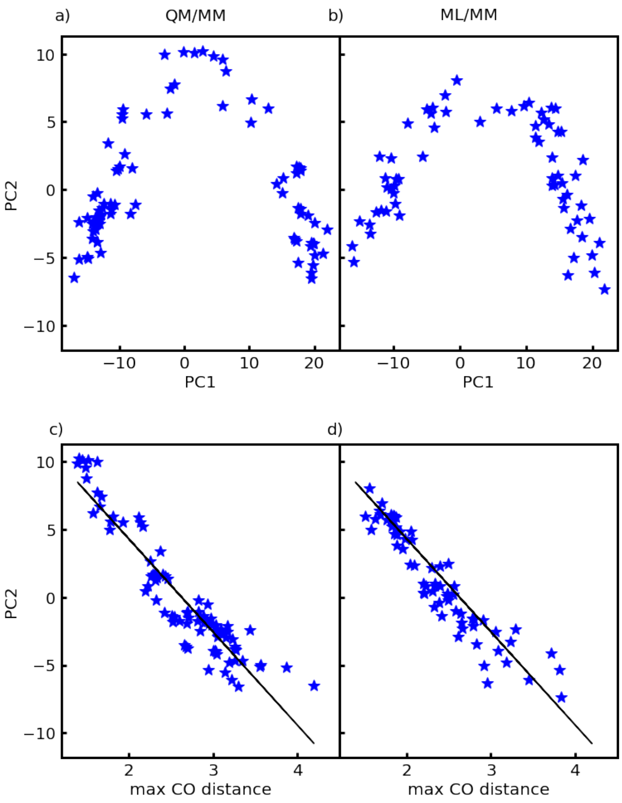

Figure S20: Projections of hopping geometries between states S_1 and S_0. a) Projection of the hopping geometries from the QM/MM reference simulations onto PC1 and PC2. b) Projection of hopping geometries from the ML/MM simulations with the 100 % random split model no. 1 onto the same principal components as in a). c) and d) show the same projections as in a) and b) with PC1 substituted by the max CO distance. In c) a linear least squares fit was applied to the QM/MM reference geometries. This line is used for comparison in Fig. SS21.

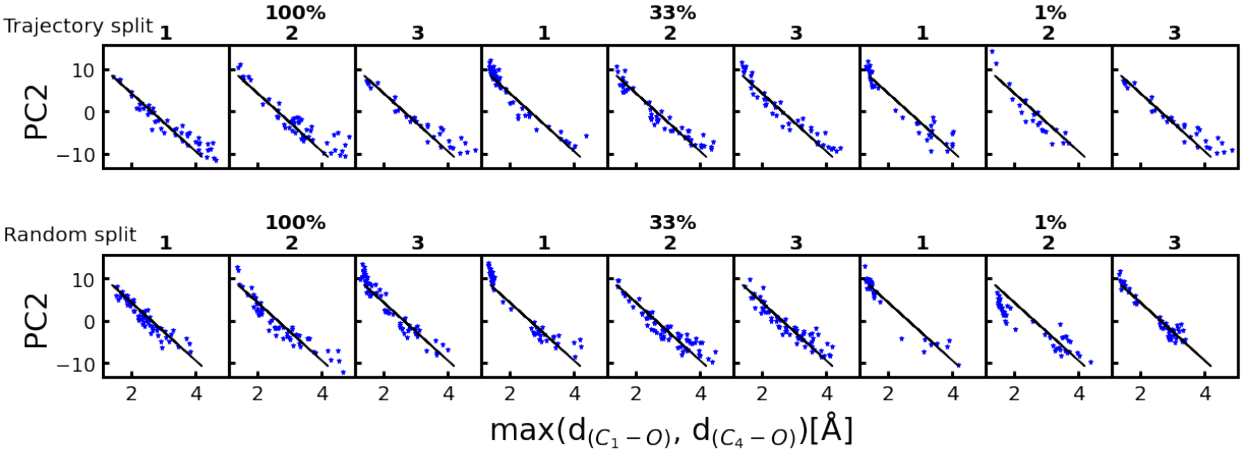

Figure S21: Projections of the hopping geometries from all ML/MM simulations onto the maximum C-O distance and the PC2 from the QM/MM data. The upper row shows the geometries obtained from trajectories generated with the split by trajectory models starting with 100 % of the data followed by 33 % and 1 %. The second row shows the same for the models trained on random splits. The black line represents a linear fit projections of the QM/MM reference data.

4.2.2 $S_2 \rightarrow S_1$

We applied the same workflow to the hopping geometries collected for hops between the states S_2 and S_1. Performing PCA on the Coulomb matrices of these geometries yields a PC1 that explains 74 % and a PC2 that explains 15 % of the total variance. All other PCs contribute less than 10 % each. Figure S22 shows the QM/MM and ML/MM hopping geometries projected onto the first two PCs obtained from the QM/MM geometries, in panel a and b respectively. The geometries form one cluster with two clear outliers for the QM/MM simulations. Both of the outliers stem from the same trajectory and represent a hop from the S_1 to the S_2 with a very low likelihood and a hop back one time step later, where one of the C-O bonds is breaking. PC1 and PC2 are again mostly determined by the two C-O bonds. As all other geometries for this transition are extremely similar (see Fig. 8a in the main text), the PCs are extremely sensitive to changes in these bonds.

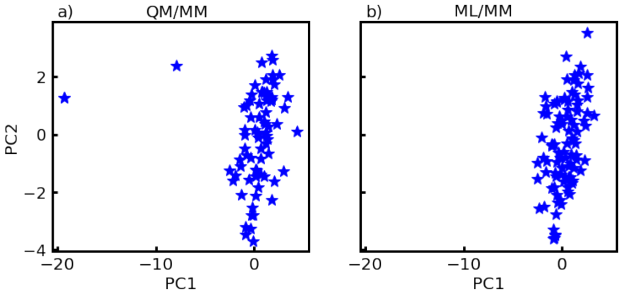

Figure S22: Projections of hopping geometries between states S_2 and S_1. a) Projection of the hopping geometries from the QM/MM reference simulations onto PC1 and PC2 (different components than in Fig. SS20). b) Projection of hopping geometries from the ML/MM simulations with the 100 % random split model no. 1 onto the same principal components as in a).

Figure S21 shows the hopping geometries between S_2 and S_1 for all ML/MM simulations projected into the first and second principal components obtained from the PCA of the reference QM/MM hopping geometries between the same states. The majority of geometries form a cluster centered at (0/0). In general, we observe that the larger the disagreement between the model and the QM/MM dynamics with respect to population curves and lifetimes, the more points deviate from this cluster and spread to lower PC1 values. Since the transition from the S_2 to the S_1 happens shortly after the excitation in the QM/MM simulations, furan cannot be very distorted in the hopping frames. Therefore, the slower the relaxation from the S_2 to the S_1 in the ML/MM dynamics, the more time there is for structural changes and the greater the deviation from the cluster at (0/0) we observe. Although this is a general trend, the absence of "outliers" does not mean that all hops occurred at the beginning of the simulation.

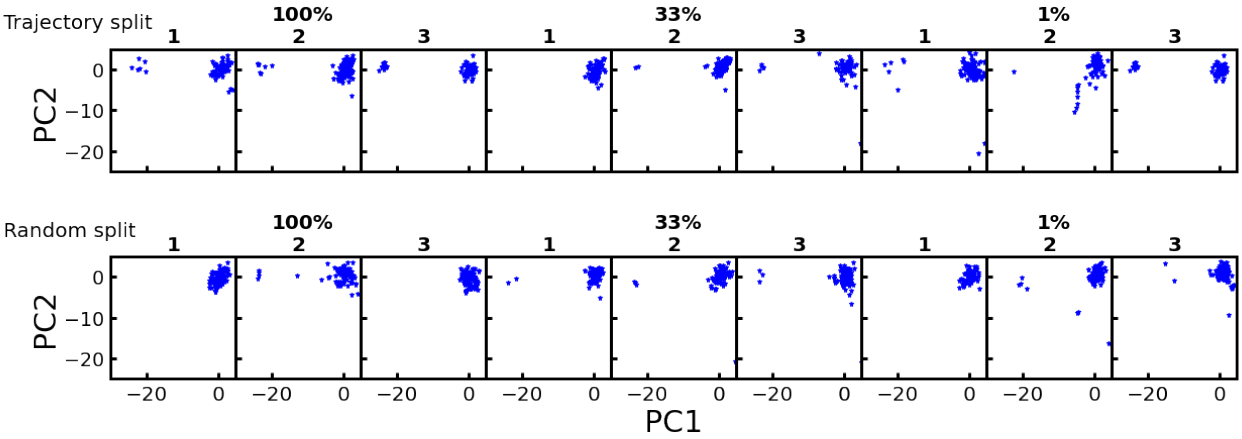

Figure S23: Projections of the hopping geometries from all ML/MM simulations onto PC1 and PC2 obtained from the QM/MM data. The upper row shows the geometries obtained from trajectories generated with the split by trajectory models starting with 100 % of the data followed by 33 % and 1 %. The second row shows the same for the models trained on random splits.

5 Additional Figures

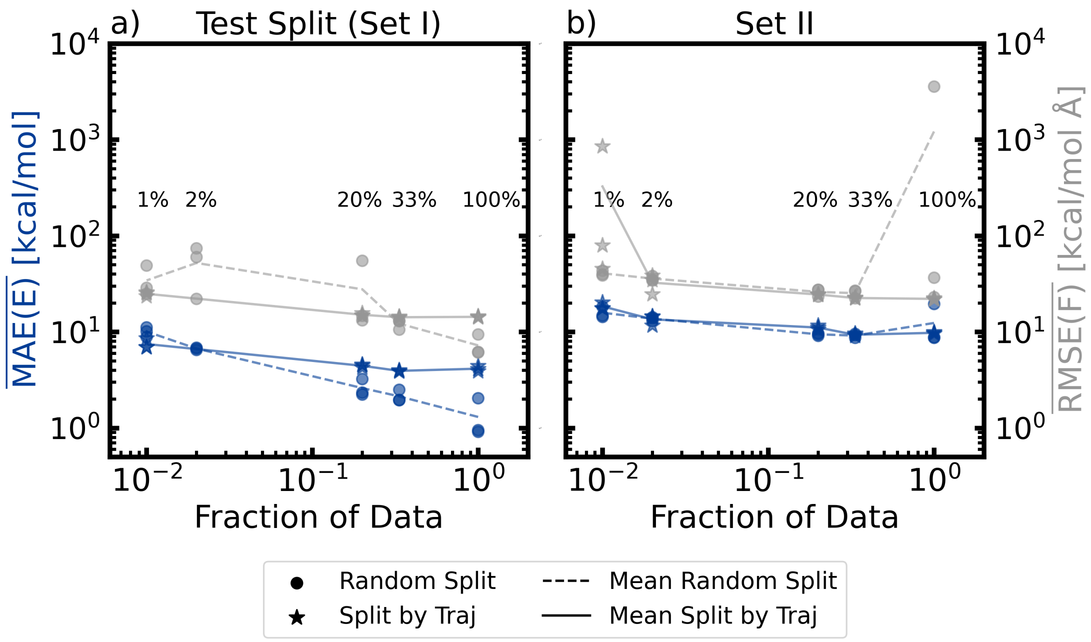

Figure S24: This is the version of Fig. 6 from the main text including the outliers in subfigure b). Change in model test performance on a) the test set taken from set I and b) trajectories from set II, as a function of the fraction of data used during model training. Results of "Random Split" models are shown as circles, "Split by Trajectory" as stars. Mean absolute error (MAE) of the energy and root mean square error (RMSE) in the forces are shown in blue and grey, respectively. All values correspond to averages over all five electronic states, indicated by the bars over MAE and RMSE. The three symbols for each combination of split type and fraction of data correspond to the three models trained with different random initializations To guide the eye, lines connect the averages of the models with identical hyperparameters.

References

(1) Fuji, T.; Suzuki, Y.-I.; Horio, T.; Suzuki, T.; Mitrić, R.; Werner, U.; Bonačić-Koutecký, V. Ultrafast photodynamics of furan. *Journal of Chemical Physics* **2010**, *133*, 234303.

(2) Rupp, M.; Tkatchenko, A.; Müller, K.-R.; von Lilienfeld, O. A. Fast and accurate modeling of molecular atomization energies with machine learning. *Physical Review Letters* **2012**, *108*, 1–5.
